# Efficacy and safety of lenvatinib combined with PD-1/PD-L1 inhibitors plus Gemox chemotherapy in advanced biliary tract cancer

**DOI:** 10.3389/fimmu.2023.1109292

**Published:** 2023-01-18

**Authors:** Chengpei Zhu, Jingnan Xue, Yunchao Wang, Shanshan Wang, Nan Zhang, Yanyu Wang, Longhao Zhang, Xu Yang, Junyu Long, Xiaobo Yang, Xinting Sang, Haitao Zhao

**Affiliations:** Department of Liver Surgery, State Key Laboratory of Complex Severe and Rare Diseases, Peking Union Medical College Hospital, Chinese Academy of Medical Sciences and Peking Union Medical College (CAMS & PUMC), Beijing, China

**Keywords:** advanced biliary tract cancer, lenvatinib, PD-1 inhibitor, PD-L1 inhibitor, Gemox chemotherapy

## Abstract

**Background:**

Lenvatinib combined with anti-PD-1 antibodies and systemic chemotherapy has demonstrated a relatively high antitumor activity for intrahepatic cholangiocarcinoma in phase 2 clinical trials. However, its efficacy and safety in advanced biliary tract cancer (BTC) has not been reported in a real-world study.

**Methods:**

Patients with advanced BTC who received lenvatinib combined with PD-1/PD-L1 inhibitors plus oxaliplatin and gemcitabine (Gemox) chemotherapy were retrospectively screened. The overall survival, progression-free survival, objective response rate, disease control rate, clinical benefit rate, and safety were evaluated.

**Results:**

Fifty-seven patients with advanced BTC were included in the study. The median follow-up time was 15.1 (95% CI: 13.6–19.7) months. The median overall survival and progression-free survival were 13.4 (95% CI: 10.0–NA), and 9.27 (95% CI: 7.1–11.6) months, respectively. The objective response rate, disease control rate and clinical benefit rate were 43.9% (95% CI: 31.8%–56.7%), 91.2% (95% CI: 81.1%–96.2%), and 73.7% (95% CI: 61.0%–83.4%), respectively. Subgroup analysis revealed that the first-line treatment group had a longer median progression-free survival (12.13 vs. 6.77 months, P<0.01) and median overall survival (25.0 vs. 11.6 months, P=0.029) than the non-first-line treatment group. Moreover, three patients underwent conventional surgery after treatment. All patients (100%) experienced adverse events, and 45.6% (26/57) experienced grade 3 or 4 adverse events. The most commonly observed grade 3 or 4 adverse events was myelosuppression (7/57, 12.3%). No grade 5 adverse events were reported.

**Conclusion:**

Lenvatinib combined with PD-1/PD-L1 inhibitors and Gemox chemotherapy represents an effective and tolerable treatment option in patients with advanced BTC.

## Introduction

Biliary tract cancer (BTC), one of the most aggressive malignant tumors, is characterized by high heterogeneity and a complex tumor microenvironment (1). BTC can be divided into gallbladder cancer (GBC) and intrahepatic and extrahepatic cholangiocarcinoma according to anatomical location (2). Owing to the difficulty of early diagnosis and easy development of chemotherapy resistance, its morbidity and mortality are increasing yearly, and its prognosis is poor (3, 4). Surgical resection is a good treatment option for early BTC, however, most patients are unresectable or have distant metastasis at the time of diagnosis.

Although the combination of gemcitabine and cisplatin (GC) has been established as first-line therapy for advanced BTC, the objective response rate (ORR) is low (1, 5–7). In the ABC-002 study, the ORR in BTC was 21%–37% (5). Further, the ABC-06 study results suggest that FOLFOX chemotherapy (folinic acid, fluorouracil, and oxaliplatin) provides improved overall survival (OS) in patients with advanced BTC, compared to active symptom control (6.2 vs. 5.3 months, respectively) (8). The overall effect of chemotherapy is limited, and once patients develop resistance or disease progression, treatment options are limited.

With the progress in research on immune checkpoint inhibitors, their combination with chemotherapy has become a treatment option for BTC, with ORR reaching 55.6% (9–11). Zhou et al. conducted a phase 2 clinical trial including 30 pathologically confirmed patients with advanced intrahepatic cholangiocarcinoma (ICC) treated with oxaliplatin and gemcitabine (Gemox) chemotherapy in combination with the anti-PD1 antibody toripalimab and lenvatinib as first-line therapy. The median progression-free survival (PFS) was 10.0 months, and the median OS was not reached, with an ORR of 80% (12). Li recently reported the results of tislelizumab combined with lenvatinib and Gemox regimen for conversion therapy of potentially resectable locally advanced BTC; the ORR and disease control rate (DCR) were 56% and 92%, respectively (13). These studies suggest that immunotherapy combined with targeted therapy and systemic chemotherapy is an effective treatment option in advanced BTC to achieve a relatively good ORR.

Based on preclinical data, we conducted a retrospective study to evaluate the safety and efficacy of lenvatinib combined with PD-1/PD-L1 inhibitors and Gemox chemotherapy for patients with advanced BTC in a real-world study. We believe that PD-1/PD-L1 inhibitors plus lenvatinib and Gemox chemotherapy may be an encouraging therapeutic regimen for patients with advanced BTC.

## Materials and methods

### Study population

**Figure 1:**
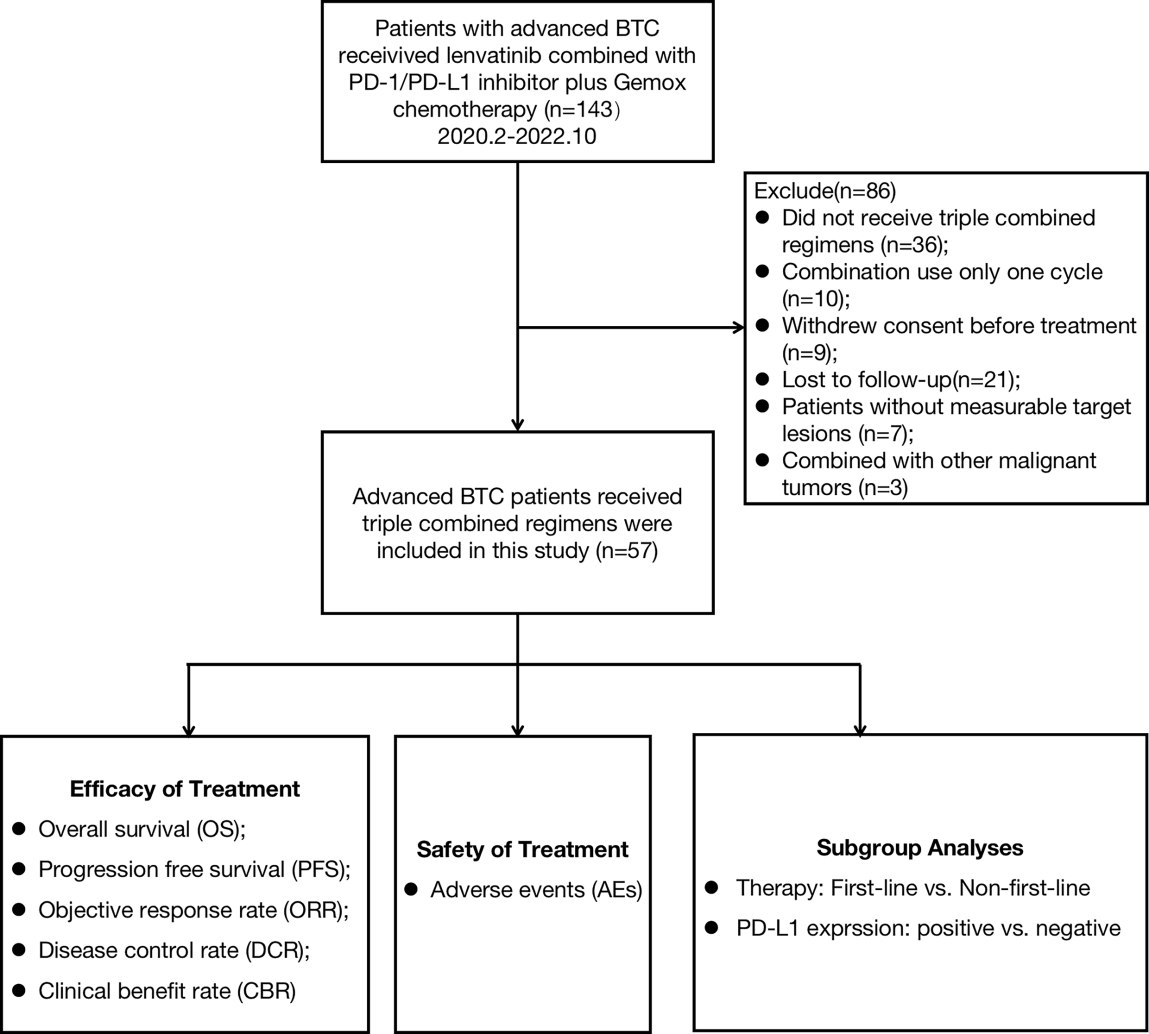
Flow diagram of study design.

Between February 2020 and October 2022, patients with advanced BTC who received lenvatinib combined with PD-1/PD-L1 inhibitors and Gemox chemotherapy at Peking Union Medical College Hospital (PUMCH) were enrolled in this study. The primary eligibility criteria included histologically confirmed BTC and at least one measurable tumor lesion according to the RECIST v1.1 criteria (14). A total of 143 patients were screened and enrolled: 36 patients did not receive immunotherapy combined with targeted therapy and chemotherapy, 10 patients received only one cycle of combination treatment, 9 patients withdrew consent before treatment, 21 patients were lost to follow-up, 7 patients had no lesions to evaluate, and 3 patients had other additional malignant tumors (**Figure 1**). Finally, 57 patients were enrolled in this study. The baseline characteristics of the 57 patients are summarized in **Table 1**. Demographic, Eastern Cooperative Oncology Group (ECOG), Child-Pugh score, cancer antigen 19-9 (CA19-9), tumor subtype, differentiated histology, disease stage, site of metastases, PD-L1 expression, previous treatment regimens for non-first-line subgroup, and the type of anti-PD-1/PD-L1 antibodies were compiled and recorded. The study was approved by the Institutional Review Board (IRB) and Ethics Committee (EC) of PUMCH (No. JS-1391).

**Table 1:** Baseline characteristics of the entire and subgroup study population.

Parameters	Total (n=57)	First-line Group (n=25)	Non-first-line Group (n=32)	P-value*
*Age, years (median, IQR)*	59 (51-64)	59 (51-68)	58 (51-62.5)	
≥ 60	25 [43.9%]	10 [40.0%]	15 [46.9%]	0.604
< 60	32 [56.1%]	15 [60.0%]	17 [53.1%]	
Sex, n [%]				
Female	20 [35.1]	7 [28.0]	13 [40.6]	0.322
Male	37 [64.9]	18 [72.0]	19 [59.4]	
ECOG performance, n [%]				
0	22 [38.6]	10 [40.0]	12 [37.5]	0.937
1	31 [54.4]	13 [52.0]	18 [56.3]	
2	4 [7.0]	2 [8.0]	2 [6.2]	
Child–Pugh score, n [%]				
A	37 [64.9]	18 [72.0]	19 [59.4]	0.322
B	20 [35.1]	7 [28.0]	13 [40.6]	
*CA19-9, U/mL (median, IQR)*	210.6 (28.2-946)	209 (24.3-800)	202 (32.39-1000)	
≥ 200	27 [47.4%]	12 [48.0%]	15 [46.9%]	0.665
< 200	23 [40.3%]	11 [44.0%]	12 [37.5%]	
NA	7 [12.3%]	2 [80%]	5 [15.6%]	
HBV infection, n [%]	5 [8.8]	1 [4.0]	4 [12.5]	0.26
Tumor subtype, n [%]				
ICC	30 [52.6]	14 [56.0]	16 [50.0]	0.777
GBC	18 [31.6]	8 [32.0]	10 [31.3]	
ECC	9 [15.8]	3 [12.0]	6 [18.7]	
Differentiated histology, n [%]				
Poor	22 [38.6]	9 [36.0]	13 [40.6]	0.862
Moderate	24 [42.1]	10 [40.0]	14 [43.8]	
Well	5 [8.8]	3 [12.0]	2 [6.2]	
NA	6 [10.5]	3 [12.0]	3 [9.4]	
TNM stage, n [%]				
III	21 [36.8]	10 [40.0]	11 [34.4]	0.662
IV	36 [63.2]	15 [60.0]	21 [65.6]	
Site of metastases, n [%]				
Intrahepatic	43 [75.4]	20 [80.0]	23 [71.9]	0.479
Lymph nodes	43 [75.4]	19 [76.0]	24 [75.0]	0.931
Lung	8 [14.0]	5 [20.0]	3 [9.4]	0.252
Bone	4 [7.0]	2 [8.0]	2 [6.2]	0.797
Others	6 [10.5]	2 [8.0]	4 [12.5]	0.583
PD-L1 expression, n [%]				
Positive	8 [14.0]	4 [16.0]	4 [12.5]	0.473
Negative	28 [49.1]	10 [40.0]	18 [56.3]	
NA	21 [36.8]	11 [44.0]	10 [31.2]	
*Therapy-line, n [%]*		–	–	
First-line	25 [43.9]	–	–	
Non-first-line	32 [56.1]	–	–	
*Previous treatment regimens for non-first-line subgroup, n [%]*	n=32	–	n=32	
Systemic chemotherapy	24 [75.0]	–	24 [75.0]	
Targeted therapy	10 [31.3]	–	10 [31.3]	
Transarterial chemoembolization	4 [12.5]	–	4 [12.5]	
Radical surgery resection	1 [3.1]	–	1 [3.1]	
Regional radiotherapy	6 [18.8]	–	6 [18.8]	
Type of anti-PD-1/PD-L1 antibodies, n [%]				
Toripalimab	24 [42.1]	9 [36.0]	15 [46.9]	0.177
Pembrolizumab	11 [19.3]	7 [28.0]	4 [12.5]	
Tislelizumab	12 [21.1]	3 [12.0]	9 [28.1]	
Durvalumab	10 [17.5]	6 [24.0]	4 [12.5]	

*First-line Group vs. Non-first-line Group.

ECOG, Eastern Cooperative Oncology Group; CA19-9, cancer antigen 19-9; TNM, tumor node metastasis classification; HBV, hepatitis type B virus; ICC, intrahepatic cholangiocarcinoma; ECC, extrahepatic cholangiocarcinoma; GBC, gallbladder cancer.

### Treatment

Lenvatinib was administered orally at a dose of 12 mg (for patients with body weight ≥ 60 kg) or 8 mg (for patients with body weight < 60 kg) once a day. The PD-1 dose included a fixed dose of 200 mg (240 mg for toripalimab) every 3 weeks or a fixed dose of 3 mg/kg body weight every 3 weeks. The recommended dose of durvalumab is 10 mg/kg body weight intravenously (IV) every 3 weeks. The Gemox chemotherapy regimen was administered as 1 g/m² gemcitabine on days 1 and 8, and 100 mg/m² oxaliplatin on day 1and then every three weeks by IV injection for six cycles. Gemox chemotherapy was stopped after 6 cycles, and immunotherapy plus targeted therapy was continued until disease progression appeared.

### Outcome assessment and PD-L1 expression evaluation

The clinical objective response was measured using the RECIST v1.1 criteria (14) and evaluated by professional radiologists at the PUMCH. To assess tumor growth rate and treatment response, computed tomography (CT)/magnetic resonance imaging or positron emission tomography CT images were regularly evaluated. PFS, OS, ORR, DCR, and clinical benefit rate (CBR) were used to assess treatment efficacy. CBR was defined as the proportion of patients with a radiologically confirmed objective response (CR or PR) or SD for > 6 months (15). Safety assessments and grading were recorded from the electronic medical records of patients or collected by the investigators using the Common Terminology Criteria for Adverse Events (version 5.0) as a reference. Subgroup analyses were also performed. PD-L1 expression was evaluated by immunohistochemistry of formalin-fixed, paraffin embedded tumor specimens, and PD-L1 overexpression was defined as more than 5% positive expression in tumor cells.

### Statistical analysis

The data from the analysis cutoff date (October 15, 2022) in this study were used to generate summaries of baseline characteristics, therapeutic efficacies, and AEs. PFS, and OS were estimated using the Kaplan–Meier method, and the comparisons were analyzed using the log-rank test. Hazard ratios of each clinicopathological feature for PFS and OS were estimated using the Cox proportional hazard model. To compare the individual variables, the t-test, Mann–Whitney U test, χ^2^ test, and Fisher’s exact test were performed, as appropriate. Statistical significance was set at P < 0.05. Statistical analyses were performed using SPSS 25 software and R-4.2.0 (https://www.r-project.org/).

## Results

### Baseline characteristics of the 57 patients

A total of 57 patients with BTC who received lenvatinib combined with PD-1/PD-L1 inhibitors and Gemox chemotherapy were included in the study. The demographic and baseline characteristics of the 57 patients are summarized in **Table 1**. At the time of initial treatment, the median age was 59 years, and 64.9% of the patients were men. Fifty-three (93.0%) patients had an ECOG performance status of 0–1, and 37 (64.9%) patients had Child-Pugh stage A. At baseline, the median CA19-9 level was 210.6 U/mL. Five (8.8%) patients had a history of hepatitis B infection. There were 30 (52.6%) patients with ICC, 9 (15.8%) with extrahepatic cholangiocarcinoma (ECC), and 18 (31.6%) with GBC. In total, 22 (38.6%) patients had poorly differentiated histology, and 8 (14.0%) had positive PD-L1 expression. Before treatment, most patients had metastatic cancer in the liver (43/57, 75.4%), lymph nodes (43/57, 75.4%), lungs (8/57, 14.0%), bone (4/57, 7.0%), and others organs (6/57, 10.5%), and 36 patients (63.2%) had TNM stage IV disease. The patients had received different types of PD-1/PD-L1 inhibitors, 24 (42.1%) were treated with toripalimab regimen, 11 (19.3%) with pembrolizumab regimen, 12 (21.1%) with tislelizumab regimen, and 10 (17.5%) with durvalumab. Thirty-two (56.1%) patients received lenvatinib combined with PD-1/PD-L1 inhibitors and Gemox chemotherapy as the non-first-line therapy. Non-first line therapy refers to the treatment of advanced BTC after the failure of first-line treatment. Among 32 patients who received non-first-line treatment, 24 (75.0%) of them had already received systemic chemotherapy, 10 (31.3%) had received targeted therapy, 4 (12.5%) had received transarterial chemoembolization, 6 (18.8%) had received regional radiotherapy, and 1 (3.1%) had received radical surgical resection (**Table 1**).

### Treatment and efficacy

**Figure 2:**
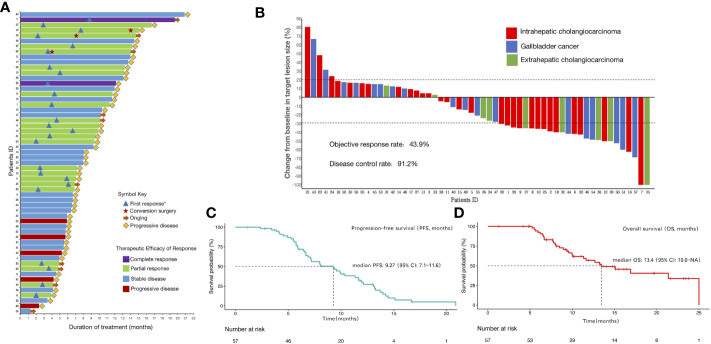
Therapeutic efficacy and treatment distribution of lenvatinib combined with PD-1/PD-L1 inhibitors plus Gemox chemotherapy in patients with advanced biliary tract carcinoma **(A)**Duration of patients’ treatments. **(B)**Maximum percentage change in the sum of the diameters of the target lesions from baseline. **(C)**Kaplan-Meier estimation of progression-free survival of the entire cohort. **(D)**Kaplan–Meier estimation of overall survival of the entire cohort. *First response was defined as the first time assessed as partial or complete response.

The median duration of treatment of lenvatinib combined with PD-1/PD-L1 inhibitors and Gemox chemotherapy was 8.0 (IQR:5.7–12.0) months. The duration of treatment for all patients is shown in **Figure 2A**. The median duration of follow-up was 15.1 (IQR, 13.6–19.7) months. Until the last follow-up date, 10 patients had no disease progression and were still receiving triple combined therapy or targeted therapy combined with immunotherapy maintenance. Three patients underwent conversion surgery after treatment, including two patients (Patient ID 16 and 22) who received triple combined therapy as first-line treatment and one (Patient ID 38) as second-line treatment. Patients 38 and 22 are currently receiving maintenance lenvatinib with PD-1/PD-L1 inhibitors durvalumab, and tislelizumab, respectively. Patient 16 had disease progression 36 days after undergoing conversion surgery.

All patients underwent complete radiological evaluation. Overall, 35 (61.4%) patients had a decrease in tumor size from baseline (**Figure 2B**). Twenty-five (43.9%) patients achieved an objective response. Of these 25 patients, 23 (40.4%) showed PR and two (3.5%) achieved CR. In total, 27 (47.4%) patients exhibited SD, and five (8.8%) patient exhibited PD. Therefore, the overall radiologically confirmed ORR was 43.9% (95% CI:31.8–56.7%) and the DCR was 91.2% (95% CI:81.1–96.2%) (**Table 2**, **Figure 2B**).

**Table 2:** Therapeutic efficacy of response and survival outcomes in entire cohort and subgroup study.

Therapeutic response assessment	Entire cohort (n = 57)	First-line Group (n=25)	Non-first-line Group (n=32)	P-value*
Objective response rate (ORR, n, %, 95% CI)	25, 43.9 (31.8-56.7)	16, 64 (44.5-79.8)	9, 28.1 (15.6-45.4)	0.007
Complete response (CR, n, %)	2 (3.5)	2 (8)	0	0.103
Partial response (PR, n, %)	23 (40.4)	14 (56)	9 (28.1)	0.033
Stable disease (SD, n, %)	27 (47.4)	8 (32)	19 (54.3)	0.04
Progressive disease (PD, n, %)	5 (8.8)	1 (4)	4 (12.5)	0.26
Disease control rate (DCR, n, %, 95% CI)	52, 91.2 (81.1-96.2)	24, 96 (80.5-99.3)	28, 87.5 (71.9-95.0)	0.26
Clinical benefit rate (CBR, n, %, 95% CI)	42, 73.7 (61.0-83.4)	21, 84 (65.4-93.6)	21, 65.6 (48.3-79.6)	0.118
Median progression free survival (mPFS, months, 95% CI)	9.27 (7.1–11.6)	12.13 (9.4–16.7)	6.77 (5.93–10.4)	0.0027
6 months PFS (%, 95% CI)	71.8 (60.7-85.0)	–	–	–
12 months PFS (%, 95% CI)	29.6 (19.2-45.5)	–	–	–
Median overall survival (mOS, months, 95% CI)	13.4 (10.0–NA)	25.0 (15.07–NA)	11.6 (8.47–NA)	0.029
6 months OS (%, 95% CI)	92.5 (85.6-99.8)	–	–	–
12 months OS (%, 95% CI)	57.0 (44.5-73.1)	–	–	–
18 months OS (%, 95% CI)	40.5 (27.0-60.8)	–	–	–

*First-line Group vs. Non-first-line Group.

The survival outcomes of the enrolled patients were investigated. For the entire cohort, the median PFS and OS were 9.27 (95% CI:7.1–11.6) months (**Figure 2C**) and 13.4 (95% CI:10.0–NA) months (**Figure 2D**), respectively. The 6-month PFS and OS rates were 71.8% (95% CI:60.7–85.0%) and 92.5% (95% CI:85.6–99.8%), respectively (**Table 2**). We further determined CBR in all patients. Impressively, the CBR in all 57 patients was 73.7% (95% CI:61.0–83.4%) (**Table 2**).

### Subgroup analyses

**Figure 3:**
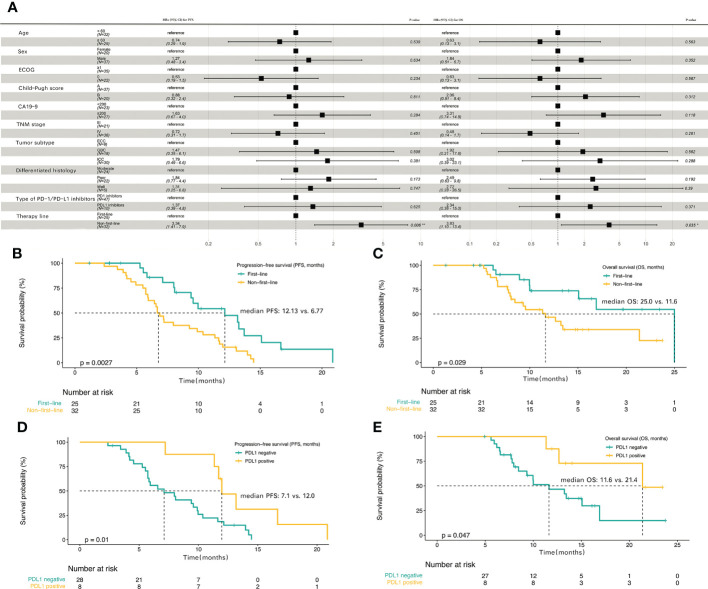
The progression-free survival and overall survival in subgroup analyses **(A)** Subgroup analyses of progression-free survival (PFS) and overall survival (OS) in the entire cohort. Kaplan-Meier plot for PFS **(B)** and OS **(C)** based on first-line treatment group compared with the non-first-line treatment group. Kaplan-Meier plot for PFS **(D)** and OS **(E)** based on PD-L1 positive expression group compared with PD-L1 negative expression group.

*Post hoc* subgroup analyses of prespecified baseline factors are presented in a forest plot (**Figure 3A**). There was no difference in the treatment effect between the subgroups, except for the therapy line. The baseline characteristics of first-line treatment compared with non-first-line treatment group are summarized in **Table 1**, and no significant differences were found between the two groups in baseline information. When patients were stratified by therapy line, Kaplan–Meier survival curve and log-rank test analysis demonstrated that patients with first-line treatment had a longer median PFS (12.13 vs. 6.77 months, P<0.01; **Figure 3B**, **Table 2**), and significantly longer OS (25.0 vs. 11.6 months, P=0.029; **Figure 3C**, **Table 2**) compared with the non-first-line treatment group. For the entire cohort, although only 36 individuals had definitive PD-L1 expression, there was a significant survival difference for these patients between the positive and negative PD-L1 expression groups (**Table S1**). Patients expressing PD-L1 (positive PD-L1 expression) had a longer median PFS (12.0 vs. 7.1 months, P = 0.01; **Figure 3D**) and a longer median OS (21.4 vs.11.6 months, P = 0.047; **Figure 3E**) compared with those not expressing PD-L1 (negative).

### Safety

**Figure 4:**
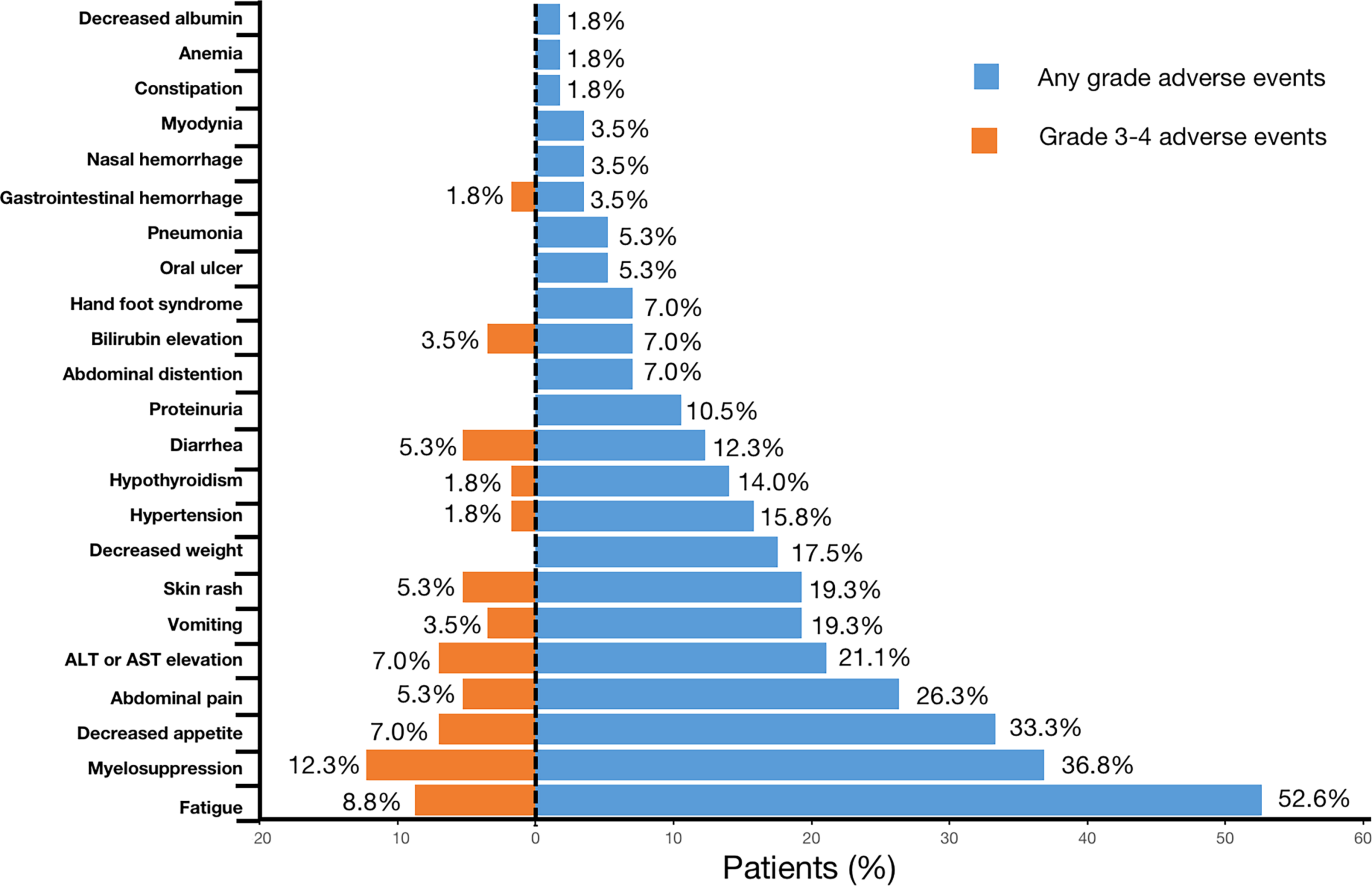
Frequency of any grade and grade 3/4 adverse events.

AEs were reported in all 57 (100%) patients throughout the treatment. No grade 5 AE occurred, and only 3.5% (2/57) of the patients experienced grade 4 AEs (bilirubin elevation and myelosuppression). For severe AEs (SAEs), 45.6% (26/57) of patients had ≥grade 3 AEs (**Table 3**, **Figure 4**). The most common AEs (of any grade) were fatigue (30/57, 52.6%), myelosuppression (21/57, 36.8%), and decreased appetite (19/57, 33.3%). The most common grade 3 or 4 SAEs were myelosuppression (7/57, 12.3%), fatigue (5/57, 8.8%), decreased appetite (4/57, 7.0%), and ALT or AST elevation (4/57, 7.0%). Most AEs that occurred during triple combination therapy were safe, well tolerated, and controlled.

**Table 3:** Commonly observed adverse events.

Adverse events (AEs)	Any grade, n (%)	Grade 3, n (%)	Grade 4, n (%)
Fatigue	30 (52.6)	5 (8.8)	0
Myelosuppression	21 (36.8)	6 (10.5)	1 (1.8)
Decreased appetite	19 (33.3)	4 (7.0)	0
Abdominal pain	15 (26.3)	3 (5.3)	0
ALT or AST elevation	12 (21.1)	4 (7.0)	0
Vomiting	11 (19.3)	2 (3.5)	0
Skin rash	11 (19.3)	3 (5.3)	0
Decreased weight	10 (17.5)	0	0
Hypertension	9 (15.8)	1 (1.8)	0
Hypothyroidism	8 (14.0)	1 (1.8)	0
Diarrhea	7 (12.3)	3 (5.3)	0
Proteinuria	6 (10.5)	0	0
Abdominal distention	4 (7.0)	0	0
Bilirubin elevation	4 (7.0)	1 (1.8)	1 (1.8)
Hand foot syndrome	4 (7.0)	0	0
Oral ulcer	3 (5.3)	0	0
Pneumonia	3 (5.3)	0	0
Gastrointestinal hemorrhage	2 (3.5)	1 (1.8)	0
Nasal hemorrhage	2 (3.5)	0	0
Myodynia	2 (3.5)	0	0
Constipation	1 (1.8)	0	0
Anemia	1 (1.8)	0	0
Decreased albumin	1 (1.8)	0	0

ALT, alanine aminotransferase; AST, aspartate aminotransferase.

## Discussion

BTC is a lethal and highly malignant tumor with a low response rate and a poor prognosis. Compared to the published literature, this is the first study to assess the efficacy and safety of PD-1/PD-L1 inhibitors plus lenvatinib with Gemox chemotherapy for patients with advanced BTC in the real world. This study also included the largest sample size yet to be reported for the treatment of BTC with triple combined therapy. This therapeutic regimen demonstrated excellent antitumor activity in BTC, with a median PFS of 9.27 months (95% CI:7.1–11.6), median OS of 13.4 months (95% CI:10.0–NA), ORR of 43.9% (95% CI:31.8–56.7), DCR of 91.2% (95% CI:81.1%–96.2%), and CBR of 73.7% (95% CI:61.0%–83.4%). All patients (100%) experienced AEs; however, no grade 5 SAEs were reported, and 45.6% (26/57) of the patients experienced grade 3 or 4 AEs. The most common AE was fatigue (30/57, 52.6%), and the most common grade 3 or 4 AE was myelosuppression (7/57, 12.3%). Subgroup analysis suggested that the use of this therapeutic regimen as first-line treatment for patients with BTC could result in better PFS (12.13 vs. 6.77 months, P = 0.0027) and OS (25.0 vs. 11.6 months, P = 0.029) compared with the non-first-line treatment subgroup.

A previous phase 2 clinical trial reported an ORR as high as 80% for patients with ICC treated with Gemox chemotherapy combined with the anti-PD1 antibody toripalimab and lenvatinib (12), but in our real-world study of patients with BTC, the ORR was only 43.9%. Our study has shown that 56.1% of the patients belonged to the non-first-line treatment, and the prompt application of the scheme has been confirmed for the first time to be aggravating, and the effect after application solutions is relatively poor. The ORR in the first-line treatment group were significantly higher than non-first-line treatment group in this study (64.0% vs. 28.1%, P=0.007), when subgroup analyses were performed. Another study reported an ORR of 56% for tislelizumab combined with lenvatinib and Gemox in potentially resectable locally advanced BTC (13). A study of PD-1 inhibitors combined with hepatic arterial infusion in BTC reported an ORR of only 11.5% (16), suggesting that targeted therapy and immunotherapy combined with systemic chemotherapy have good efficacy in BTC. Drug resistance is one of the reasons that limit the efficacy of tumor immunotherapy. Combining drugs with different mechanisms of action may overcome multiple drug resistance mechanisms. Most chemotherapeutic agents are acting through their direct cytotoxic effects without considering the impact on the immune system (17). Studies have shown that chemotherapy can increase the response to immunotherapy by increasing the immunogenicity of tumor cells or by inhibiting the immunosuppressive circuit (18, 19). Chemotherapy enhances the effect of immunotherapy mainly through the following mechanisms (17). First, cytotoxic chemotherapy, such as platinum, gemcitabine and other drugs, can significantly reduce myeloid-derived suppressor cells (MDSCs) by activating caspase-8-dependent apoptosis and selectively depleting monocytes/macrophages, thereby inhibiting anticancer immunity. Second, cytokines produced by cytotoxic chemotherapy damage to cancer cells can enhance the recruitment of antigen presenting cells (APCs), promote the phagocytosis of dendritic cells (DCs) on stressed cancer cells, and induce the secretion of other proinflammatory cytokines. In addition, small molecule drugs, such as epigenetic modulators, can up-regulate antigen processing, presentation mechanisms and costimulatory molecules through gene expression modification, and can also induce the production of cytokines to enhance the response to immunotherapy. Finally, patients with chemotherapy-resistant could respond to rechallenge with chemotherapy after anti-PD-1 therapy. Targeted drugs can eliminate cancer cells through direct anti-tumor activity and immunogenic cell death (ICD), which can not only reduce the number of cells targeted and destroyed by immune cells, but also eliminate immunosuppressive factors and improve the effect of immunotherapy (20, 21). Vascular endothelial growth factor receptor (VEGFR) is expressed on activated and memory T cells. The VEGF-VEGFR pathway plays a key role in almost all immune cell subsets, and can inhibit TCR-dependent activation of T cells and inhibit the cytotoxic activity of T cells. VEGF can also induce the accumulation of MDSCs (17). Lenvatinib is a multi-targeted tyrosine kinases inhibitor, which targets VEGFR1-3. Lenvatinib participates in the process of immune response by playing a role in the VEGF-VEGFR pathway (22, 23). Therefore, early use of targeted therapy and immunotherapy combined with systemic chemotherapy is recommended for patients who can tolerate it. The other reason is the difference in tumor subtypes for inconsistent ORR. Li suggested a better outcome for GBC and ICC than for ECC (13). Although the subgroup analysis of tumor types did not differ among ICC, GBC, and ECC (**Figures S1A, B**), and ICC and GBC types accounted for 84.2% in this study (**Table 1**), the influence of disease cannot be excluded due to the small sample size. There is still some controversy regarding whether PD-L1 expression can be used as a biomarker for targeted therapy combined with immunotherapy (24–26). In our study, only 36 individuals had definitive PD-L1 expression, the positive rate of PD-L1 expression was only 22.2% (8/36), however, the median OS (21.4 vs. 11.6 months, P=0.047), median PFS (12.0 vs. 7.1 months, P=0.01), and ORR (75.0% vs. 35.7%, P=0.049) in PD-L1 positive group were higher compared with PD-L1 negative group (**Table S1**, **Figures 3D, E**). Previous study reported that PD-L1 expression can be used as a potential marker for predicting immunotherapy effectiveness (25, 27). More studies are needed to confirm whether PD-L1 expression is related to the efficacy of targeted therapy and immunotherapy combined with chemotherapy in BTC.

Although targeted therapy and immunotherapy combined with chemotherapy resulted in more AEs, they were generally manageable. In our study, although all patients experienced varying degrees of AEs, no grade 5 AEs were observed. Approximately 45.6% (26/57) of the patients experienced ≥ grade 3 AEs and 3.5% (2/57) experienced grade 4 AEs. The most frequent AEs were fatigue (n=30; 52.6%), myelosuppression (n=21; 36.8%), and decreased appetite (n=19; 33.3%). The incidence of bone marrow suppression was higher in our study than that reported in other studies (28), which may be due to the use of chemotherapy in this study. Myelosuppression is a common adverse reaction to chemotherapy (5, 29, 30). In a cohort study of pembrolizumab combined with lenvatinib, the incidence of grades 3 and 4 AEs was 59.5% and 3.1%, respectively (28), suggesting that the addition of chemotherapy to targeted therapy and immunotherapy does not increase the occurrence of AE in present study.

This study had some limitations. First, this was a single-center real-world study with small sample size; thus, the results should be interpreted with caution. In the future, more large-sample, multicenter, prospective studies are needed to confirm these results. Second, several immunotherapeutic drugs were used, including immunotherapy containing anti-PD-1 and anti-PD-L1regimens. Although there was no difference in the subgroup analysis in this study (**Figures S1C, D**), and triple combined therapy with different immunotherapy agents all showed good ORR (12, 13), prospective studies with a single drug are still needed to confirm the differences in outcomes using different drug choices. Third, in the subgroup analysis, there was no difference in the tumor classification of BTC; however, few studies have reported that targeted therapy combined with immunotherapy and chemotherapy may be more effective in GBC and ICC than ECC (13), which needs to be confirmed by future studies on different pathological types. Finally, this is a retrospective, single-arm study, which lacks a control group with standard treatment regimens including chemotherapy. Prospective clinical trials are needed to remedy the shortcomings of the current study. Although this study has its limitations, as a real-world study, it can be used as a reference for the design of subsequent clinical research and the selection of clinical treatment strategies.

## Conclusions

PD-1/PD-L1 inhibitors combined with lenvatinib and Gemox chemotherapy are effective, safe, and well-tolerated in advanced BTC. This combination therapy may prolong the survival of patients with advanced BTC as first-line treatment compared with non-first-line treatment. Further research with larger prospective cohorts is required.

## Data availability statement

The original contributions presented in the study are included in the article/**Supplementary Material**. Further inquiries can be directed to the corresponding authors.

## Ethics statement

The studies involving human participants were reviewed and approved by Institutional Review Board (IRB) and Ethics Committee (EC) of PUMCH. The patients/participants provided their written informed consent to participate in this study.

## Author contributions

All authors contributed to the study conception, design and data collection. Material preparation and analysis was performed by CZ, JX and YCW. CZ, JX and YCW wrote the first draft of the manuscript and all authors commented on the subsequent versions of the manuscript. All authors contributed to the article and approved the submitted version.

## Supplementary material

Supplementary Figure 1 Subgroup analyses based on other factors. Kaplan-Meier plot for PFS **(A)** and OS **(B)** based on tumor subtype. Kaplan-Meier plot for PFS **(C)** and OS **(D)** based on type of anti-PD-1/PD-L1 antibodies.
