# Supplementary figures and images for: Efficacy and safety of lenvatinib combined with PD-1/PD-L1 inhibitors plus Gemox chemotherapy in advanced biliary tract cancer
Source: Front Immunol. 2023 Jan 18;14:1109292. doi: 10.3389/fimmu.2023.1109292 (PMC9889821; doi:10.3389/fimmu.2023.1109292)

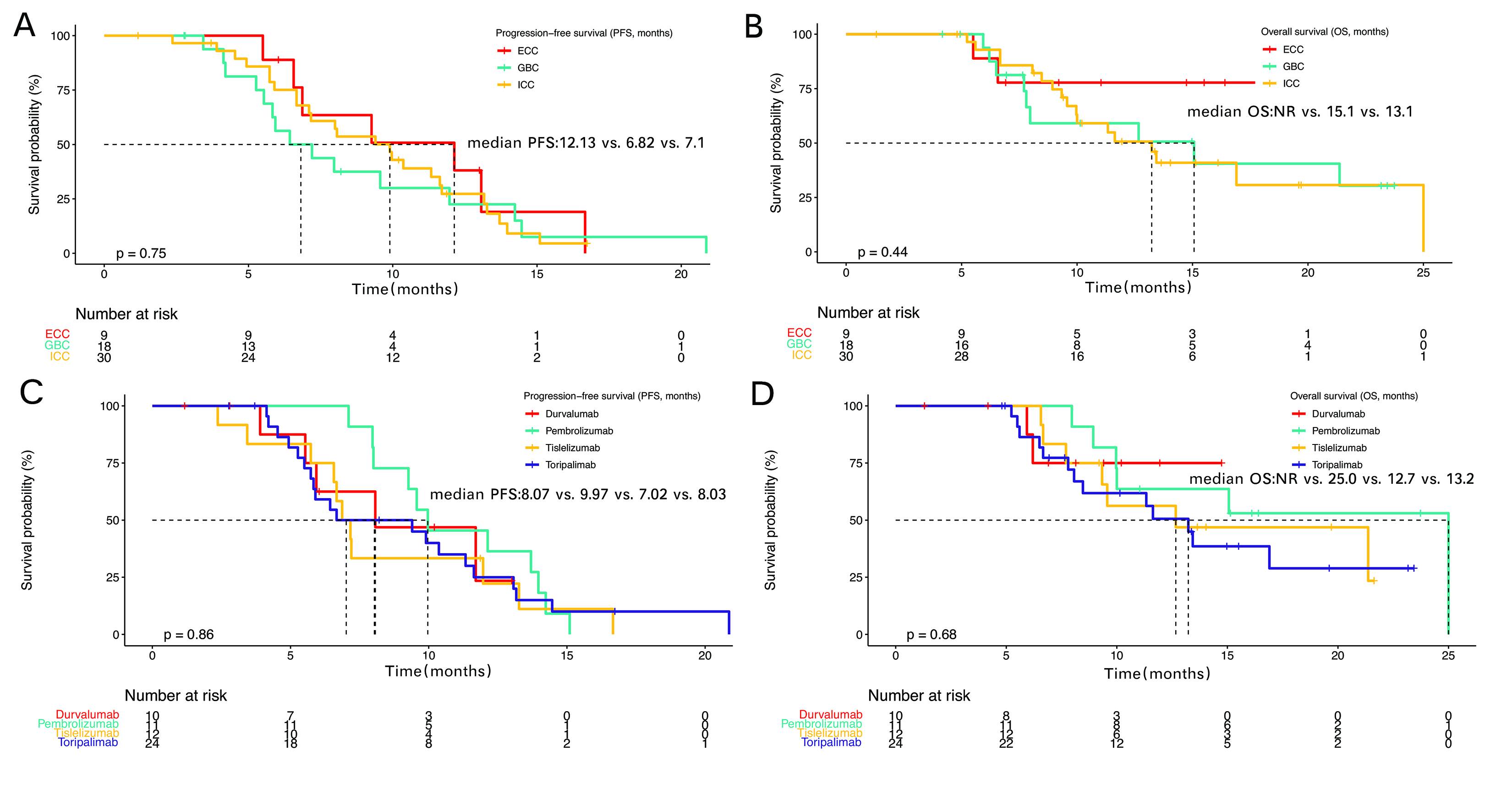

Supplement: Supplementary Figure 1 — Subgroup analyses based on other factors. Kaplan-Meier plot for PFS (A) and OS (B) based on tumor subtype. Kaplan-Meier plot for PFS (C) and OS (D) based on type of anti-PD-1/PD-L1 antibodies. [file Image_1.tif]
