# Supplementary material for: Efficacy and safety of lenvatinib combined with PD-1/PD-L1 inhibitors plus Gemox chemotherapy in advanced biliary tract cancer
Source: Front Immunol. 2023 Jan 18;14:1109292. doi: 10.3389/fimmu.2023.1109292 (PMC9889821; doi:10.3389/fimmu.2023.1109292)
Supplement: Supplementary file 2 [file DataSheet_1.docx]

Supplementary Material

Efficacy and safety of lenvatinib combined with PD-1/PD-L1 inhibitors plus Gemox chemotherapy in advanced biliary tract cancer

Chengpei Zhu^1†^, Jingnan Xue^1†^, Yunchao Wang^1†^, Shanshan Wang^1^, Nan Zhang^1^, Yanyu Wang^1^, Longhao Zhang^1^, Xu Yang^1^, Junyu Long^1^, Xiaobo Yang^1^, Xinting Sang^1*^, Haitao Zhao^1*^

^1^Department of Liver Surgery, State Key Laboratory of Complex Severe and Rare Diseases, Peking Union Medical College Hospital, Chinese Academy of Medical Sciences and Peking Union Medical College (CAMS & PUMC), Beijing, China.

^†^These authors contributed equally to this work and should be considered as co-first authors.

*** Correspondence:**

Haitao Zhao,

Department of Liver Surgery, State Key Laboratory of Complex Severe and Rare Diseases, Peking Union Medical College Hospital, Chinese Academy of Medical Sciences and Peking Union Medical College (CAMS & PUMC), #1 Shuaifuyuan, Wangfujing, Beijing 100730, China.

Email: zhaoht@pumch.cn

Xinting Sang,

Department of Liver Surgery, State Key Laboratory of Complex Severe and Rare Diseases, Peking Union Medical College Hospital, Chinese Academy of Medical Sciences and Peking Union Medical College (CAMS & PUMC), #1 Shuaifuyuan, Wangfujing, Beijing 100730, China.

Email: sangxt@pumch.cn

# Supplementary Figures and Tables

## Supplementary Figures

**Fig.S1 Subgroup analyses based on other factors.** Kaplan-Meier plot for PFS (A) and OS (B) based on tumor subtype. Kaplan-Meier plot for PFS (C) and OS (D) based on type of anti-PD-1/PD-L1 antibodies.

## Supplementary Tables

**Table S1 Therapeutic response for positive and negative PD-L1 expression group**

| Therapeutic response assessment | Entire cohort (n = 57) | Positive PD-L1 expression group (n=8) | Negative PD-L1 expression group (n=28) | P-value* |
| --- | --- | --- | --- | --- |
| ORR (n, %, 95% CI) | 25, 43.9% (31.8-56.7%) | 6, 75%  (40.9-92.9%) | 10, 35.7%  (20.7-54.2%) | **0.049** |
| CR (n, %) | 2 (3.5%) | 0 | 1 | 0.497 |
| PR (n, %) | 23 (40.4%) | 6 | 9 | **0.03** |
| SD (n, %) | 27 (47.4%) | 2 | 14 | 0.209 |
| PD (n, %) | 5 (8.8%) | 0 | 4 | 0.257 |
| DCR (n, %, 95% CI) | 52, 91.2% (81.1-96.2%) | 8, 100% | 24, 85.7%  (68.5-94.3%) | 0.257 |
| CBR (n, %, 95% CI) | 42, 73.7% (61.0-83.4%) | 8, 100% | 17, 60.7%  (42.4-76.4) | **0.033** |
| mPFS (months, 95% CI) | 9.27 (7.1–11.6) | 12.0 (11.70–NA) | 7.1 (5.73–9.97) | **0.01** |
| mOS (months, 95% CI) | 13.4 (10.0–NA) | 21.4 (12.67–NA) | 11.6 (8.47–NA) | **0.047** |

*Positive PD-L1 expression group vs. Negative PD-L1 expression group
